# TACTICS VR Stroke Telehealth Virtual Reality Training for Health Care Professionals Involved in Stroke Management at Telestroke Spoke Hospitals: Module Design and Implementation Study

**DOI:** 10.2196/43416

**Published:** 2023-12-07

**Authors:** Steven Maltby, Carlos Garcia-Esperon, Kate Jackson, Ken Butcher, James W Evans, William O'Brien, Courtney Dixon, Skye Russell, Natalie Wilson, Murielle G Kluge, Annika Ryan, Christine L Paul, Neil J Spratt, Christopher R Levi, Frederick Rohan Walker

**Affiliations:** 1 Centre for Advanced Training Systems The University of Newcastle Newcastle Australia; 2 Hunter Medical Research Institute New Lambton Heights Australia; 3 School of Biomedical Sciences & Pharmacy College of Health, Medicine & Wellbeing The University of Newcastle Callaghan Australia; 4 John Hunter Hospital New Lambton Heights Australia; 5 NSW Agency for Clinical Innovation St Leonards Australia; 6 School of Clinical Medicine University of New South Wales Sydney Australia; 7 Department of Neurosciences Gosford Hospital Gosford Australia; 8 School of Medicine and Public Health College of Health, Medicine & Wellbeing The University of Newcastle Callaghan Australia; 9 John Hunter Health & Innovation Precinct New Lambton Heights Australia

**Keywords:** virtual reality, technology, medical education, telehealth, stroke management, stroke workflow

## Abstract

**Background:**

Stroke management in rural areas is more variable and there is less access to reperfusion therapies, when compared with metropolitan areas. Delays in treatment contribute to worse patient outcomes. To improve stroke management in rural areas, health districts are implementing telestroke networks. The New South Wales Telestroke Service provides neurologist-led telehealth to 23 rural spoke hospitals aiming to improve treatment delivery and patient outcomes. The training of clinical staff was identified as a critical aspect for the successful implementation of this service. Virtual reality (VR) training has not previously been used in this context.

**Objective:**

We sought to develop an evidence-based VR training module specifically tailored for stroke telehealth. During implementation, we aimed to assess the feasibility of workplace deployment and collected feedback from spoke hospital staff involved in stroke management on training acceptability and usability as well as perceived training impact.

**Methods:**

The TACTICS VR Stroke Telehealth application was developed with subject matter experts. During implementation, both quantitative and qualitative data were documented, including VR use and survey feedback. VR hardware was deployed to 23 rural hospitals, and use data were captured via automated Wi-Fi transfer. At 7 hospitals in a single local health district, staff using TACTICS VR were invited to complete surveys before and after training.

**Results:**

TACTICS VR Stroke Telehealth was deployed to rural New South Wales hospitals starting on April 14, 2021. Through August 20, 2023, a total of 177 VR sessions were completed. Survey respondents (n=20) indicated a high level of acceptability, usability, and perceived training impact (eg, accuracy and knowledge transfer; mean scores 3.8-4.4; 5=strongly agree). Furthermore, respondents agreed that TACTICS VR increased confidence (13/18, 72%), improved understanding (16/18, 89%), and improved awareness (17/18, 94%) regarding stroke telehealth. A comparison of matched pre- and posttraining responses revealed that training improved the understanding of telehealth workflow practices (after training: mean 4.2, SD 0.6; before training: mean 3.2, SD 0.9; *P*<.001), knowledge on accessing stroke telehealth (mean 4.1, SD 0.6 vs mean 3.1, SD 1.0; *P*=.001), the awareness of stroke telehealth (mean 4.1, SD 0.6 vs mean 3.4, SD 0.9; *P*=.03), ability to optimally communicate with colleagues (mean 4.2, SD 0.6 vs mean 3.7, SD 0.9; *P*=.02), and ability to make improvements (mean 4.0, SD 0.6 vs mean 3.5, SD 0.9; *P*=.03). Remote training and deployment were feasible, and limited issues were identified, although uptake varied widely (0-66 sessions/site).

**Conclusions:**

TACTICS VR Stroke Telehealth is a new VR application specifically tailored for stroke telehealth workflow training at spoke hospitals. Training was considered acceptable, usable, and useful and had positive perceived training impacts in a real-world clinical implementation context. Additional work is required to optimize training uptake and integrate training into existing education pathways.

## Introduction

### Background and Rationale

Ischemic stroke is a leading (and increasing) cause of adult-acquired disability worldwide [1]. Longer time to reperfusion treatment has severe and negative impacts for patients with ischemic stroke [2], with a progressively increased likelihood of poor patient outcome with every hour of delay before reperfusion treatment is started [3]. Variability in clinical care is a major contributor to delayed treatment [4], particularly in rural areas, and there are numerous challenges in delivering best practice stroke management in rural contexts in Australia [5] and worldwide [6]. One significant contributor to delays at rural sites is the nature of the workforce, which is typically more junior, nonspecialist, and transient than the workforce at metropolitan hospitals, as well as having less access to continuing professional development training [7-9]. There is extensive clinical trial evidence to inform best practice stroke workflow to deliver reperfusion therapies (eg, thrombolysis and endovascular thrombectomy). However, additional research is needed to inform the implementation of effective workflow processes and training in real-world clinical settings, particularly in rural areas.

To address variability in stroke care in rural settings, telestroke networks have been developed in Australia [10,11] and worldwide [12,13], where rural spoke hospitals are supported by comprehensive centers. Locally, in New South Wales (NSW) in Australia, we first initiated a telestroke network in 2013 with a single rural hospital, which expanded over subsequent years to include 5 rural sites linked to a single comprehensive stroke center. The telestroke service aims to provide urgent access to a stroke neurologist, improving access to reperfusion therapies as well as outcomes at rural sites. To date, the service has documented increased thrombolysis treatment rates [14] and highlighted the value of multimodal computed tomography (CT) imaging for stroke diagnosis in rural areas [15]. The successful initial implementation of the local telestroke service provided pilot data [14] and is a cornerstone of the current statewide NSW Telestroke Service, which has undertaken a progressive rollout providing services to 23 rural hospitals [16,17]. The implementation of the statewide service started in March 2020, with staggered rollout across the 23 sites through June 2022. We proposed that virtual reality (VR) training to support telestroke service implementation would be useful at both existing and new sites as a complement to support existing training, new staff inductions, training refreshers for existing staff, and initial site onboarding.

Acute stroke management workflow is complex, and efficient team coordination and the sequencing of processes are challenging. The effective delivery of timely reperfusion treatment in acute ischemic stroke requires structured stroke workflow processes. Intensive quality improvement processes aimed at optimizing stroke workflow pathways by enhancing team communication and implementing site-specific processes can markedly improve reperfusion treatment delivery times [18-20], for example, the implementation of multifaceted implementation strategies via targeted quality improvement at 2 Australian comprehensive hospitals effectively reduced time to treatment [18,19]. However, site-specific stroke workflow pathways are often not applied at rural hospitals where there are gaps in local training and resourcing, transient workforces, and limited effective application of quality improvement activities. In this context, stroke telehealth services can complement local processes by providing access to expert neurologist support and implementing a structured routine for stroke workflow. Importantly, the successful integration of telestroke services requires training local staff to increase awareness, confidence, and competence in telestroke processes.

Staff education and training are critical for the delivery of evidence-based stroke management, but this is limited in rural settings. Less than two-thirds of Australian physicians and nurses involved in stroke management report having received any interactive or competency-based training, including approximately 60% of nurses and 40% of physicians [21]. Stroke management staff in regional areas, at sites with fewer annual stroke admissions (<75/year), and at sites without a dedicated stroke unit were less likely to have opportunities for professional development (63%, 62%, and 64%, respectively) [22]. Stroke workflow training is typically delivered by neurology specialists via traditional face-to-face workshops. This training happens infrequently, has high logistical complexity and costs, and cannot be readily accessed by all staff at a given rural hospital. The delivery of training via this traditional model was further impaired during the COVID-19 pandemic, when travel between sites was restricted [23]. On the basis of our previous work developing and implementing VR-based workflow training for health care staff in hyperacute stroke management in rural settings [24], we proposed VR-based training specific for stroke telehealth to support the implementation of the NSW Telestroke Service.

In medical fields, VR-based training consistently increases student engagement and motivation [25,26] and is effective for practical skills acquisition [27,28] and knowledge development [29-31]. VR technology has previously been applied in the context of both stroke management and medical education [32-34]. To date, VR applications for stroke have primarily targeted patients rather than health care professionals (eg, in the context of limb rehabilitation [35,36]). In the broader medical education context beyond stroke, most VR training applications for health care professionals have only been assessed in small pilot trials or in the context of medical school or residency training (systematic reviews and meta-analyses [37-39]).

In a nontelehealth context, we previously developed and implemented a novel fit-for-purpose VR-based stroke workflow training application called TACTICS VR Hyperacute Stroke Management [24]. During initial implementation in the first trial cluster of 7 hospitals, TACTICS VR users reported a high degree of usability, acceptability, utility, and feasibility [24]. Trainees self-reported increased confidence in their ability to make improvements in stroke management after training [24]. The application is currently actively being assessed in the Trial of Advanced CT Imaging and Combined Education Support for Drip and Ship, which is implementing a *package intervention* to support advanced CT imaging and streamlined workflow training at rural sites in 3 Australian states and will also report effects on clinical behavior and patient outcomes [40]. On the basis of the initial success of implementing TACTICS VR training in rural hospitals within a clinical trial context, we proposed the development of a second TACTICS VR module to support the implementation of the NSW Telestroke Service in this study.

### Study Objectives

This paper describes the development and implementation of TACTICS VR Stroke Telehealth, a VR training module for spoke hospital workflow processes. The platform was conceptualized to support the NSW Telestroke Service rollout and provide improved training and awareness on effective stroke management to health care professionals at spoke hospitals involved in stroke management (eg, physicians, nurses, and radiographers). The study aims to assess TACTICS VR Stroke Telehealth implementation against critical elements that typically predict the future adoption and uptake of new technologies in educational practice. This includes the feasibility of the platform as a training resource in a real-world clinical setting, specifically in rural areas. Furthermore, end user acceptance, including perceived usefulness and usability, and perceived training impacts are assessed via survey feedback.

## Methods

### Ethical Considerations

Ethics approval for the overall NSW Telestroke Service research protocol was granted by the Prince of Wales Hospital Human Research Ethics Committee. Because of the practical constraints of ethics approval being governed at a local health district level, the collection of user survey feedback was limited to a subset of sites within a single local health district (n=7) via ethics approval from the Hunter New England Human Research Ethics Committee (REGIS Ref 2019/ETH01238 and HNEHREC Ref 18/09/19/4.13) lodged with The University of Newcastle Human Research Ethics Committee (H-2019-0343). Before the deployment of TACTICS VR training, all study sites provided consent to participate. Individual user participation was voluntary, and users were able to use TACTICS VR without participating in the study. Participants were advised that their consent was implied by the completion of the surveys.

### Project Conception and Scoping

The NSW Telestroke Service was jointly funded by the NSW and Australian federal governments in 2019, with service delivery at the first rural sites starting in March 2020 [16,17]. It is a collaboration between the Prince of Wales Hospital, eHealth NSW, the NSW Agency for Clinical Innovation (ACI), and the NSW Ministry of Health in partnership with the Australian Stroke Foundation. The NSW Telestroke Service was rolled out progressively to its total coverage, with the final sites activated in June 2022, providing services to 23 rural hospitals across NSW [41].

TACTICS VR Stroke Telehealth was initially conceived through discussions among the Centre for Advanced Training Systems (ATS) at The University of Newcastle, the ACI, and the NSW Telestroke Service in 2019, informed by the development and implementation of the first TACTICS VR Hyperacute Stroke Management module within the TACTICS trial [24,40].

### Study Methodology and Protocol

#### Overview

A descriptive survey study approach was applied to the creation and deployment of TACTICS VR Stroke Telehealth. Quantitative and qualitative data were collected during the development (from May 1, 2020, through April 14, 2021) and implementation of the training module across 23 hospitals in NSW (from April 14, 2021, through August 20, 2023). A particular focus was placed on assessing elements that typically predict technology adoption, including end user acceptance (perceived usability and usefulness), training impacts, and the practical challenges of technology delivery within a real-world health care setting.

#### TACTICS VR Stroke Telehealth Module

**Figure 1:**
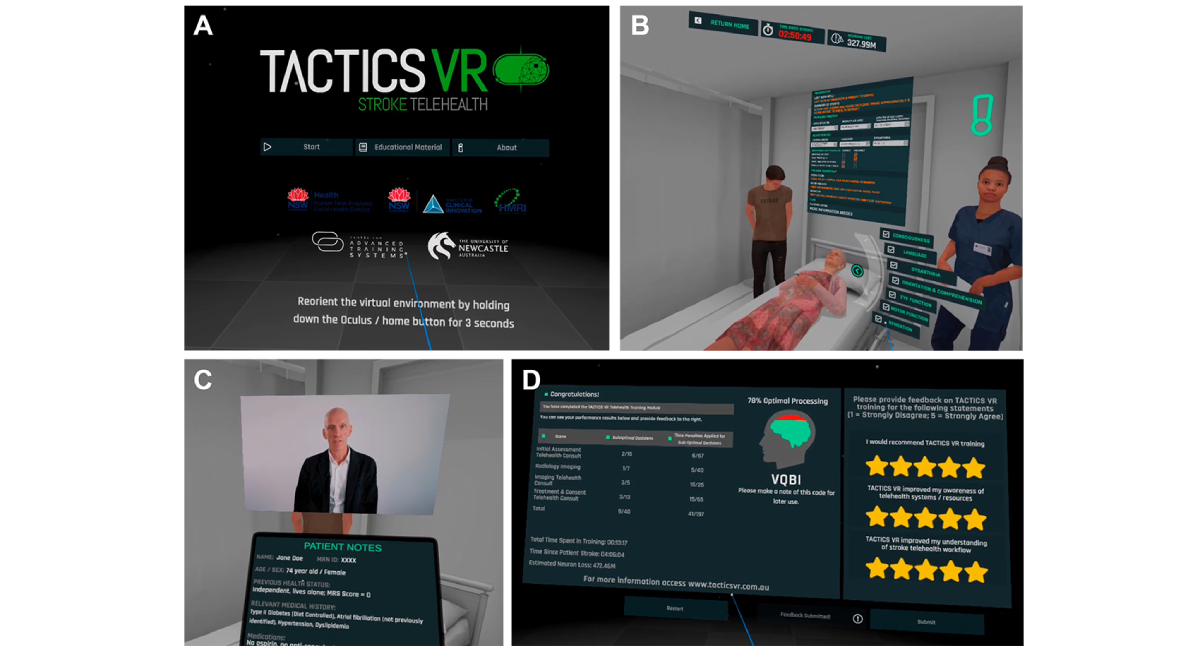
Screenshots and key features of TACTICS VR Stroke Telehealth. (A) Starting scene. (B) Representative scene, including avatars and user interactions. (C) Representative talking head video and tablet view for patient information. (D) User feedback scene.

TACTICS VR Stroke Telehealth was developed for the stand-alone Oculus Quest 2 enterprise-enabled headset (Oculus or Meta) using the Unity platform (Unity Technologies). A detailed overview of included scenes, key features, objectives, and user interactions is provided in Table 1, with representative images presented in Figure 1. Users initially enter a 3D VR infinity space containing the application menu and brief overview text about the application. Upon initiating training, users are requested to provide brief demographic information (eg, hospital location and position title) and are provided a video overview of the training module and instructions on how to use the VR technology. Users then proceed through a single real-world stroke case from the prenotification and initial assessment phase through multimodal CT imaging, consenting, treatment with thrombolysis, and patient transfer for endovascular thrombectomy. Specific emphasis is placed on 3 telehealth consults for assessment, imaging interpretation, and consenting and treatment, with all other aspects of the workflow streamlined to minimize the total length of training. Users interact with elements in the VR environment, including avatars (eg, patient and nurse), equipment (eg, telephone and computer), and question and answer prompts to gather case information and prompt active decision-making. Suboptimal decisions result in immediate feedback (via text or audio) and time penalties, providing an element of gamification. For each user interaction, the accuracy was calculated as the number of suboptimal decisions selected (including both suboptimal decisions made and optimal decisions missed) as a percentage of total possible decisions. Time penalties for each suboptimal decision (or missed optimal decision) were arbitrarily determined through context expert feedback based on the perceived priority and impact that the specific decision would have on real-world workflow times (ie, suboptimal decisions deemed more serious were assigned larger time penalties). A stroke clock and an estimate of neuron loss are displayed at the top of the screen to emphasize the importance of accurate and timely workflow and model the real-world consequences of delayed or inaccurate decision-making. At the end of the module, users are provided with feedback on their training, including time spent in training, errors, and time penalties.

**Figure 2:**
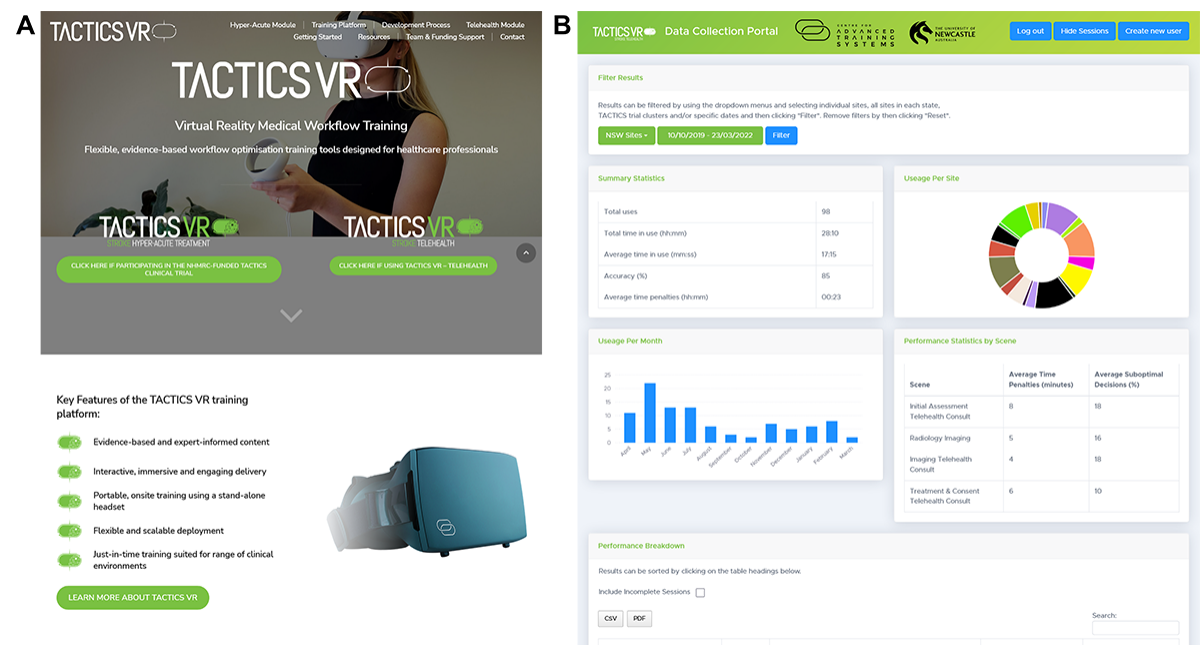
TACTICS VR training platform. The preexisting TACTICS VR platform was updated to integrate (A) a publicly accessible website for user support and educational resources as well as (B) an automated backend analytics portal for the collection of training uptake and user performance data.

TACTICS VR Stroke Telehealth was integrated into the existing TACTICS VR training platform, which includes a public-facing supporting website [42] and backend automated database and password-protected reporting environment (Figure 2). A detailed overview of the TACTICS VR training platform has been published previously [24]. In this study, the public website was updated with a streamlined design to include information about the VR training module, the development process, funding sources, and contributors and stakeholders [42]. The database and reporting environment were duplicated from the first TACTICS VR module and updated to reflect locations, user interactions, and scenes from the TACTICS VR Stroke Telehealth module to support the automated tracking of training implementation. The developed training module was endorsed by the Australasian Stroke Academy.

**Table 1:** TACTICS VR Stroke Telehealth scenes, key features, objectives, and user interactions.

Scene	Features	Objectives	User interactions
Start and introduction	VR^a^ infinity space Contributor and funding information Demographics capture (location, position title, department, experience, and previous TACTICS VR use)	Initiation of training Demographics capture Overview of training application (objectives and functionality)	Navigation buttons Q&A^b^: demographics Talking head video
Prehospital notification and initial assessment	VR ED^c^ environment Patient assessment (case details, symptoms, previous function, and history)	Familiarization with stroke assessment processes and resources Clarification of critical data required	Avatars: patient, next of kin, paramedic, and nurse Q&A: workflow processes and team interactions
Telehealth consult 1: initial assessment	Telephone consultation Communication of critical data Coordination of local team and imaging staff Patient transfer to imaging department	Familiarization with telestroke processes Workflow decision-making and team coordination	Telephone interaction: telestroke neurologist
Radiology and imaging	Virtual radiology suite with CT^d^ machine	Clear communication to initiate imaging Timing of telehealth follow-up	Avatars: patient and radiographer
Telehealth consult 2: imaging	Virtual web conferencing communication Patient transfer to ED or resuscitation suite	Interpretation of imaging Treatment decision-making	Audiovisual: imaging with neurologist commentary Q&A: workflow processes, treatment decision, and team coordination
Telehealth consult 3: consent and treatment	Virtual ED or resuscitation suite with workstation on wheels	Familiarization with consenting process Treatment administration Considerations for patient EVT^e^ transfer	Avatars: patient, next of kin, and nurse
Feedback	User performance (time, errors, and penalties)	User receives feedback on performance User provides feedback on training utility	User feedback on training (based on a scale ranging from 1 star to 5 stars)

^a^VR: virtual reality.

^b^Q&A: question and answer.

^c^ED: emergency department.

^d^CT: computed tomography.

^e^EVT: endovascular thrombectomy.

#### Study Participants and Recruitment

Stroke coordinators at spoke hospitals were requested to encourage any relevant health care staff involved in stroke management at their site to use TACTICS VR Stroke Telehealth training. All health care staff who tested the application were invited to participate in the data collection regarding its utility and training impact. Health care professionals from any background (eg, physicians, nurses, and radiographers) involved in stroke care and as identified by the stroke telehealth coordinator, were encouraged to explore TACTICS VR and provide automated anonymous feedback within the headset. Additional structured pre-post survey data were collected from this population at 7 spoke sites, with corresponding ethics approvals.

#### Measures: Self-Report and Survey Data

Self-report data (qualitative and quantitative in nature) were collected both automatically within the headset for all users and via separate structured pre-post surveys deployed at specific locations, with corresponding ethics approvals.

Within the TACTICS VR Stroke Telehealth module, basic demographics information and user feedback (location, position title, relative stroke management experience, previous use of TACTICS VR, and user ratings) is automatically collected via user prompts within the headset and automatically transferred via Wi-Fi to the password-protected reporting environment. These data are presented as TACTICS VR *user data* throughout this manuscript.

Additional structured survey responses were collected from trainees at the 7 spoke sites before and after TACTICS VR Stroke Telehealth training. Surveys collected more detailed feedback on training usability, usefulness, and perceived training impact. All survey data are referred to as *TACTICS VR survey response data* provided by *respondents* throughout this manuscript.

The administered surveys were generated by the research team and adapted from previous studies to accommodate the limited availability and time constraints of health care staff. Informed by the technology acceptance model [43-45], the survey items and domains focused on perceived acceptability, usability, usefulness, and training impacts and included individual items from validated scales, where appropriate [46,47].

Items within the pretraining survey aimed to collect relevant demographic information (eg, specialty area, position title, previous stroke telehealth experience, and VR experience), general expectations of VR-based training, and anticipated learnings. Items within the posttraining survey aimed to collect feedback on TACTICS VR Stroke Telehealth training (eg, hardware comfort, design, user interactions, and areas of improvement), usability, and usefulness and perceived value. Items relating to stroke workflow awareness, knowledge, and skill confidence aligned with TACTICS VR learning objectives and were included in both pretraining and posttraining surveys to assess training impacts via pretraining survey versus posttraining survey statistical comparisons.

The full lists of questions and responses are provided in Tables S1 and S2 in Multimedia Appendix 1.

#### Data Analysis

Self-report data were summarized and presented as mean (SD) or absolute number of responses, using Prism (version 8.0; GraphPad), and every response was included.

Statistical analysis was performed using Prism (version 8.0). Comparisons between pre- and posttraining responses to matched questions were analyzed using paired Wilcoxon tests for nonparametric data. *P* values of <.05 were considered statistically significant for all analyses.

Because of the limited number of open-ended survey responses, no formal analysis of qualitative data was performed. Points that were repeatedly mentioned in the open-ended responses were identified by the study investigators, and examples are provided in the *Results* section (a full list of responses is provided in Table S2 in Multimedia Appendix 1).

## Results

### Development of TACTICS VR Stroke Telehealth

**Figure 3:**
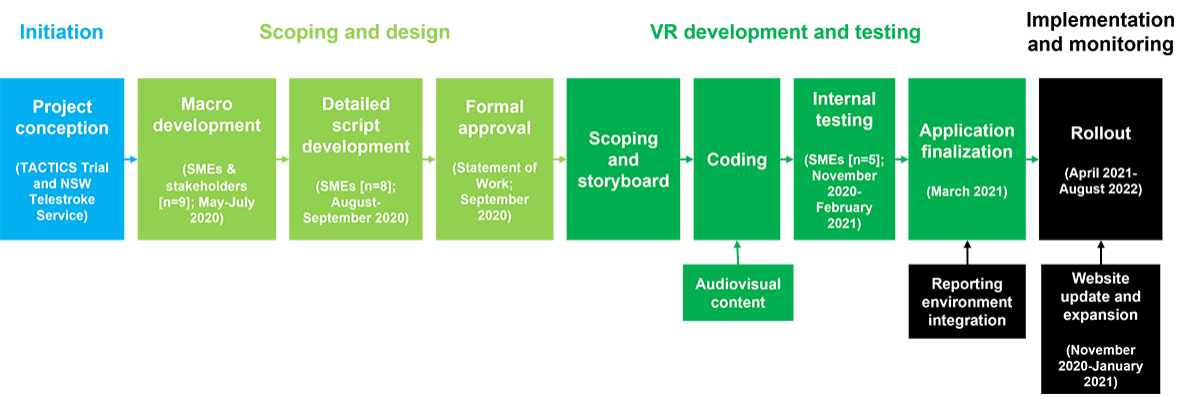
TACTICS VR Stroke Telehealth development pathway. Development occurred via a structured process from initiation through scoping and design, content development, iterative rounds of internal testing, implementation, and monitoring. NSW: New South Wales; SME: subject matter expert; VR: virtual reality.

The overall concept and philosophy for TACTICS VR Stroke Telehealth built on the preexisting TACTICS VR module and training platform [24]. TACTICS VR Stroke Telehealth was developed using a structured stepwise approach throughout (an overview schematic is presented in Figure 3). Most importantly, the development involved and actively included subject matter experts (SMEs) and stakeholders. Specifically, members from the ATS, the ACI, and the NSW Telestroke Service as well as experts, including specialist neurologists, nurses, and radiographers, were consulted and were active creators of the platform from concept to software development.

An initial *macrolevel* overview of stroke telehealth workflow was generated based on NSW Telestroke Service workflow protocols, training resources, existing education materials, and informal SME interviews. This process identified key aspects of workflow procedures and provided contextual insights. A working group of SMEs was formed to review draft script documents and provide insights into common mistakes that delay telestroke workflow. All feedback was integrated into a single statement of work, which was reviewed by stakeholders via web conferencing. Subsequent meetings with SMEs informed the development of a detailed script document specifying specific user interactions, questions and answers, user feedback, and digital media content for inclusion (eg, reference images and talking head videos). The completed workflow was summarized into a single document and formally approved by SMEs as well as members of the ATS, the ACI, and the NSW Telestroke Service. The approved statement of work was circulated to content developers to inform quotes and development. This process was chosen to ensure the development of a fit-for-purpose training tool that would be accepted by trainees and experts in the field.

Software development proceeded stepwise through four key phases: (1) VR scoping and concept creation, (2) content development and refinement, (3) internal beta testing, and (4) application finalization.

### TACTICS VR Stroke Telehealth Implementation

TACTICS VR was deployed as part of the NSW Telestroke Service, which embeds stroke coordinators at telehealth spoke sites and includes structured education and training, communications (eg, communities of practice), the deployment of technology (eg, workstation on wheels and web conferencing), and ongoing support.

Headsets were shipped out to telestroke site coordinators at 23 participating locations. Using enterprise-enabled headsets allowed the remote monitoring of activations, battery status, and the update deployment of the software, removing the management burden from the telestroke coordinators and making it the responsibility of a dedicated centralized position. Telestroke site coordinators were initially briefed and trained with the VR headset on a site-by-site basis via web conferencing and provided supporting documents (eg, a quick-access guide and an instruction manual). Site coordinators were then requested to encourage any health care staff involved in stroke management at their spoke hospital to use TACTICS VR training (in particular, members of the stroke team as well as emergency department and radiology and imaging staff [eg, physicians, nurses, and radiographers]).

A total of 177 TACTICS VR sessions were logged at 20 (87%) of the 23 NSW Telestroke Service sites between April 14, 2021, and August 20, 2023. The number of completed training sessions per site ranged from 0 to 66 (mean 7.3, SD 13.1; median 4.5).

The mean time spent in VR training was 17 minutes 14 seconds per session, equating to a total of 50 hours 48 minutes across all sessions.

### TACTICS VR User Data

TACTICS VR user data collected within the headset showed that those who used the platform primarily worked in acute stroke or neurology (94/177, 53.1%) and emergency care (63/177, 35.6%), followed by intensive care (7/177, 4%), radiology (4/177, 2.3%), stroke rehabilitation (2/177, 1.1%), and other (7/177, 4%). Users were employed as physicians (*in training*: 79/177, 44.6%; staff specialist: 25/177, 14.1%) or nurses (nurse: 50/177, 28.2%; nurse practitioner: 2/177, 1.1%), with the remainder being radiographers (4/177, 2.3%) or other (16/177, 9%). Of the 177 users, 33 (18.6%) had previously used a TACTICS VR training module. The users had a range of previous stroke management experience in terms of both total patient numbers and years, with a bimodal distribution of users reporting low or high experience (Table 2).

The mean user response accuracy was 87.2% (SD 8.3%) overall, resulting in an average of 22 (SD 19) minutes in time penalties per session. User accuracy was highest for each of the scenes relating to treatment and consent telehealth (91%), followed by each of the scenes for initial assessment telehealth consult (83%), radiology imaging (83%), and imaging telehealth consult (83%). Upon completing training, users provided positive in-headset feedback on TACTICS VR training by rating statements on a scale ranging from 1 star to 5 stars: “I would recommend TACTICS VR training” (n=127): mean 4.6, SD 0.8; “TACTICS VR improved my awareness of telehealth systems/resources” (n=126): mean 4.6, SD 0.7; and “TACTICS VR improved my understanding of stroke telehealth workflow” (n=126): mean 4.6, SD 0.7 (Table 2).

**Table 2:** TACTICS VR users have a diverse range of stroke experience and report positive training impacts of the TACTICS VR Stroke Telehealth module in the headset. For both stroke management queries, n=177; N values are included for the TACTICS VR feedback fields (n=126-127).

Stroke management experience		Response in VR^a^ headset
**Stroke management experience, n (%)**		
	<10 patients	90 (51)
	11-20 patients	27 (15)
	21-30 patients	12 (7)
	31-40 patients	7 (4)
	>40 patients	41 (23)
**Stroke management experience, n (%)**		
	0 years	51 (29)
	1-5 years	68 (38)
	6-10 years	31 (18)
	11-15 years	20 (11)
	16-25 years	5 (3)
	>25 years	2 (1)
**TACTICS VR feedback^b^, mean (SD)**		
	TACTICS VR improved my understanding of stroke telehealth workflow (n=126)	4.6 (0.7)
	TACTICS VR improved my awareness of telehealth systems and resources (n=126)	4.6 (0.7)
	I would recommend TACTICS VR training (n=127)	4.6 (0.8)

^a^VR: virtual reality.

^b^Responses collected in VR headset on a scale from 1-5 stars.

### TACTICS VR Survey Response Data

A total of 21 pretraining and 20 posttraining survey responses were received from 4 sites (from a total of 26 VR sessions across the 7 survey-eligible sites; completed between May 2021 and September 2022; 21/26, 81% response rate during the survey deployment time frame). Survey respondents primarily worked in emergency care (14/21, 67%); there was also representation from acute stroke and neurology (3/21, 14%), intensive care (1/21, 5%), and other (stroke dietitian, research, and general or acute medicine: 3/21, 14%). Of the 21 respondents, 10 (48%) were employed as nurses, 10 (48%) as physicians, and 1 (5%) as a stroke dietitian; in addition, 3 (14%) were currently involved in a training program. Respondents had a range of previous experience in both stroke management and telestroke (Table 3). Of the 21 respondents, 2 (10%) indicated that they were prone to motion sickness when asked a binary yes or no question (17/21, 81% answered *no*).

Respondents had minimal previous experience with VR technology in any context (Table 3), and 3 (14%) of the 21 respondents had previously used any TACTICS VR training module. Before completing training, respondents indicated that they were likely to use telehealth in the future (19/21, 90% answered: mean 4.2, SD 1.0; 1=strongly disagree and 5=strongly agree) and were somewhat confident in their ability to effectively assess and treat patients with stroke (21/21, 100% answered: mean 3.5, SD 0.9; Table 3). Before the training, respondents were somewhat confident in managing the technical aspects of VR training (21/21, 100%: mean 3.6, SD 0.8; Table 3) and considered VR an effective method to teach or transfer knowledge about stroke telehealth workflow (21/21, 100% answered: mean 3.8, SD 0.8; Table 3).

After completing TACTICS VR Stroke Telehealth training, respondents provided positive feedback on the hardware, user interface, and training content across a range of aspects, including comfort, enjoyment, utility, accuracy, effectiveness in transferring knowledge, realism, and the quality of feedback (mean scores ranged from 3.8 to 4.4; 1=strongly disagree and 5=strongly agree; Table 4). Furthermore, respondents agreed that TACTICS VR Stroke Telehealth training increased their confidence in stroke telehealth (18/20, 90% answered: mean 3.8, SD 0.8; 13/18, 72%: agree or strongly agree), improved their understanding of stroke telehealth (18/20, 90% answered: mean 4.2, SD 0.8; 16/18, 89%: agree or strongly agree), improved their awareness of stroke telehealth (18/20, 90% answered: mean 4.3, SD 0.6; 17/18, 94%: agree or strongly agree), and indicated that as a result of TACTICS VR Stroke Telehealth training, they were more likely to use stroke telehealth in the future (19/20, 95% answered: mean 4.0, SD 0.8; 14/19, 74%: agree or strongly agree; Table 4).

A comparison of responses to matched questions between pre- and posttraining surveys indicated that TACTICS VR training increased user confidence in the understanding of telehealth workflow processes (after training: mean 4.2, SD 0.6; before training: mean 3.2, SD 0.9; median 4, IQR 4-5 vs median 4, IQR 3-4; *P*<.001), knowledge around accessing stroke telehealth (mean 4.1, SD 0.6 vs mean 3.1, SD 1.0; median 4, IQR 4.00-4.25 vs median 3, IQR 2-4; *P*=.001), awareness of local stroke telehealth services or processes (mean 4.1, SD 0.6 vs mean 3.4, SD 0.9; median 4, IQR 4.00-4.25 vs median 4, IQR 2.50-4.00; *P*=.03), ability to optimally communicate with colleagues to enable effective treatment (mean 4.2, SD 0.6 vs mean 3.7, SD 0.9; median 4, IQR 4-5 vs median 4, IQR 3-4; *P*=.02), and ability to make improvements to how stroke care is provided (mean 4.0, SD 0.6 vs mean 3.5, SD 0.9; median 4, IQR 4-4 vs median 4, IQR 3-4; *P*=.03; Table 5). A nonsignificant trend was also noted toward increased confidence in the understanding of general workflow practices to manage patients with acute stroke (mean 4.3, SD 0.5 vs mean 3.9, SD 0.6; median 4, IQR 4-5 vs median 4, IQR 4-4; *P*=.15), noting that self-reported pretraining confidence was already relatively high. Of the 20 respondents, 3 (15%) reported that they experienced motion sickness, but all were able to complete training. None of the respondents reported technical issues using TACTICS VR Stroke Telehealth, and the majority (13/18, 72%) believed that additional TACTICS VR training modules should be developed.

When asked an open-ended question on which elements of the TACTICS VR Stroke Telehealth training module were most beneficial, respondents primarily noted the VR virtual environment, interactions or interactivity, content presentation, and the overview of the complete workflow process. Selected comments are provided herein (all responses are available in Table S2 in Multimedia Appendix 1):

- Visual environment and scans, feedback on selections made, and step-by-step process
- Explanation of telestroke workflow and input
- Easy to follow
- Instant feedback: “awesome”
- Explanations provided at each decision point
- Feedback or interaction

Respondents were also asked which elements of TACTICS VR Stroke Telehealth could be improved, and they primarily identified aspects relating to VR technology familiarity, site-specific differences in processes, and areas for future development or expansion (eg, role-specific workflow modules). Comments included the following:

- Mock scenario at beginning or instructions on how to navigate through scenarios
- Perhaps the selections could be more clear regarding only selecting the first step in parallel (ie, “checklist that you tick”)
- Role oriented (not nurse specific; “seems more medical officer”)
- Further information regarding interpreting scans, including information regarding contraindications to tissue plasminogen activator or endovascular clot retrieval (stroke treatments)

Respondents who indicated that the additional TACTICS VR training modules should be developed (13/18, 72%) were asked what additional modules would be useful. Responses included the following:

- Allied health team involvement (or physiotherapist, speech therapist, and dietitian to demonstrate the whole of team care)
- Trauma survey and cardiac thrombolysis
- Procedural skills training

**Table 3:** Pretraining survey respondents have a range of stroke management experience, limited virtual reality (VR) experience, and existing attitudes to VR training.

Stroke, telestroke, and virtual reality experience		Survey response
**Approximately how many acute stroke patients have you cared for or treated over the course of your career? n (%)**		
	Less than 10 patients	7 (33)
	11-20 patients	4 (19)
	21-30 patients	2 (10)
	31-40 patients	2 (10)
	41 patients or more	6 (29)
**Approximately how many stroke telehealth cases have you been involved in? n (%)**		
	0 patients	8 (38)
	1-4 patients	8 (38)
	5-9 patients	0 (0)
	10-14 patients	2 (10)
	≥15 patients	2 (10)
	Not applicable to my practice	1 (5)
**What is your experience with virtual reality technology? n (%)**		
	Over 100 hours of usage	0 (0)
	50-99 hours of usage	0 (0)
	10-49 hours of usage	1 (5)
	Less than 10 hours of usage	6 (29)
	No experience	14 (67)
**Pretraining confidence and attitudes^a^, mean (SD)**		
	I am likely to use telehealth services in the future	4.2 (1.0)
	I am confident in my ability to effectively assess or treat acute stroke patients	3.5 (0.9)
	I feel confident in regards to managing the technical aspect of the VR training tool	3.6 (0.8)
	I believe that VR can be an effective method to teach or transfer knowledge about stroke telehealth workflow	3.8 (0.8)

^a^Pretraining survey responses collected on 5-point Likert scale (1=strongly disagree and 5=strongly agree; n=19-21).

**Table 4:** Survey respondents provided positive feedback on TACTICS VR Stroke Telehealth training and reported perceived training benefits.

			Posttraining survey response^a^, mean (SD)
**Feedback on TACTICS VR stroke telehealth**			
	The VR hardware (eg, headset and controller) were comfortable and easy to use	4.1 (0.9)	
	The TACTICS VR Telehealth user interface was straight-forward and easy to use (eg, menu system, buttons, etc)	4.4 (0.7)	
	I enjoyed TACTICS VR Telehealth training	4.2 (0.6)	
	TACTICS VR Telehealth provided useful information to be useful for trainees and staff in the management of acute stroke	4.2 (0.4)	
	TACTICS VR Telehealth provided accurate information	4.2 (0.7)	
	TACTICS VR Telehealth was an effective tool for transferring knowledge about stroke telehealth practices	4.4 (0.5)	
	The simulation was of sufficient realism to communicate the critical aspects of workflow	3.9 (1.0)	
	The feedback provided at the end of the VR training module was constructive and useful	4.0 (0.6)	
**Perceived training impact**			
	TACTICS VR Telehealth improved my awareness of stroke telehealth approaches	4.3 (0.6)	
	TACTICS VR Telehealth improved my understanding of stroke telehealth approaches	4.2 (0.8)	
	TACTICS VR Telehealth increased my confidence in stroke telehealth workflow	3.8 (0.8)	
	As a result of TACTICS VR Telehealth training I am more likely to use stroke telehealth services in the future	4.0 (0.8)	

^a^Survey responses collected on 5-point Likert scale (1=strongly disagree and 5=strongly agree; n=17-20).

**Table 5:** Survey respondents provide positive feedback on perceived training effects regarding confidence, via comparison of posttraining versus pretraining survey responses.

Self-reported stroke management confidence	Pretraining survey response^a^, mean (SD)	Posttraining survey response^a^, mean (SD)	*P* value
I am confident in my understanding of telehealth workflow processes	3.2 (0.9)	4.2 (0.6)	<.001
I am confident in my knowledge around accessing telehealth for acute stroke assessment or treatment	3.1 (1.0)	4.1 (0.6)	.001
I am aware of local stroke telehealth services and processes	3.4 (0.9)	4.1 (0.6)	.03
I am confident in my ability to optimally communicate with my colleagues to enable effective treatment of acute stroke patients	3.7 (0.9)	4.2 (0.6)	.02
I am confident in my ability to make improvements to how acute stroke care is provided to patients presenting to this hospital	3.5 (0.9)	4.0 (0.6)	.03
I am confident in my understanding of workflow practices to manage acute stroke patients	3.9 (0.6)	4.3 (0.5)	.15

^a^Survey responses collected on 5-point Likert scale (1=strongly disagree and 5=strongly agree; n=17-21).

## Discussion

### Principal Findings

This paper describes the development and pilot implementation of TACTICS VR Stroke Telehealth, a VR-based training application specifically tailored for stroke telehealth workflow training. The TACTICS VR module was developed to address an identified training gap to support the rollout of the NSW Telestroke Service by, and with, involved stakeholders. In this study, we evaluated the acceptability, usability, and perceived training impacts of TACTICS VR Stroke Telehealth training across 20 rural NSW hospitals, including survey response data from a subset of 7 sites within a single local health district. The feedback indicated a high level of end user acceptability (including perceived usability and usefulness) among health care professionals involved in stroke management at telehealth spoke hospitals. Furthermore, the feasibility of deploying VR training in a real-world clinical setting outside of a formal clinical trial context was demonstrated, while noting that additional work is required to optimize training uptake and integration into existing training processes. TACTICS VR users provided positive feedback on application delivery and design. Survey respondents reported perceived improvements in key aspects of stroke telehealth management, including an increased understanding and awareness of telehealth workflow, knowledge around accessing stroke telehealth, an improved ability to optimally communicate with colleagues, and an increased ability to make improvements to stroke care.

Extensive participant feedback was very positive, and implementation was overall deemed feasible, but we believe that there is room for improvement in terms of overall training uptake. To encourage use and simplify deployment, we specifically developed training sessions that were relatively short (<20 minutes), included brief talking head videos emphasizing key learning objectives, and provided supporting documentation as well as a user-facing website so that training could be deployed on site without trainer support.

Although this study was not structured to objectively determine factors influencing site-specific implementation, the captured data and discussions did provide some useful insights to assess in future implementation interventions. Rural telestroke hospitals varied widely across a range of factors, including overall hospital size (from 30 to 520 beds), stroke team composition (eg, specific stroke teams vs emergency department–based or locum management, staffing numbers and positions, skill sets, and on-site vs off-site radiographers), resourcing and NSW Telestroke Service site coordinator demographics (eg, on site vs off site, preexisting members of the local team vs new appointments, clinical vs administrative background, and single site vs multisite coverage). Discussions with study investigators and site coordinators highlighted barriers relating to staffing (both trainer and trainee availability), existing attitudes and training frameworks, and competing priorities. Mapping these factors onto the theoretical domains framework highlights relevant aspects within the domains of knowledge, skills, social or professional role and identity, beliefs about capabilities, reinforcement, environmental context and resources, and social influences [48]. By applying the Behavior Change Wheel [49], we propose a range of strategies that could address these domains within local contexts, including education (eg, promoting awareness of, and practical skills in delivering, VR training; and tailored training documentation), persuasion (eg, making VR training a mandatory component of local staff orientation), incentivization, environmental restructuring (eg, local staffing and resourcing), enablement (eg, upskilling training facilitators and providing for dedicated training time), communication or marketing, and service provision (eg, facilitated training by an external service provider). In the long term, if VR training is demonstrated to be effective and sustainable in supporting clinical behavior change and improving patient outcomes, it may be appropriate to consider its integration into consensus guidelines and clinical pathways.

Future studies should seek to document enablers and barriers to VR training uptake and explore additional implementation approaches. To this end, we are currently assessing the effects of a range of approaches to TACTICS VR training uptake and sustainment, including making training mandatory, integrating training into existing continuing professional development credit systems, deploying VR headsets via roaming educators who travel among sites, providing time-limited access to hardware at individual sites on a rolling basis, delivering supported face-to-face education sessions or workshops where multiple staff members complete training in parallel (eg, 10 trainees/1-hour session), and embedding VR training within a broader staff training program with expert-supported debriefing. We anticipate that a combination of different implementation approaches and strategies, which applies a codevelopment approach to optimize training within each organization, will be useful to increase adoption across a range of rural sites with different needs.

As part of the development process, we also documented associated costs, which may be relevant to readers who are considering developing and implementing similar VR training in their specific context. Note that during the study period conversion rates ranged from approximately Aus $1 (US $0.6-$0.8). Direct costs associated with the development of TACTICS VR Stroke Telehealth training included VR application coding and updating of the existing automated analytics portal (approximately Aus $37,000; ie, ~US $24,200), expansion and redesign of the user-facing website (approximately Aus $4000; ie, ~US $2600), and hardware (VR headsets and Wi-Fi routers; approximately Aus $1800 [~US $1200] per site). Additional costs associated with project management, internal application testing, research assessment, ongoing support and maintenance, and implementation within the broader NSW Telestroke Service rollout were not specifically captured or reported.

### Comparison With Prior Work

Telestroke services improve the rates of reperfusion treatment and functional patient outcomes without increasing the rates of treatment-induced hemorrhage, although it has been noted that evidence for impacts on resource use and cost-effectiveness is limited [50,51]. As a result, patients treated through telestroke networks achieve outcomes that are similar to those managed at comprehensive stroke centers [10,12,13]. Of note, in Australia, the importance of telehealth-supported stroke care was recently recognized through its inclusion in the Australia Stroke Foundation guidelines as best practice to improve outcomes in rural settings [52]. However, the effectiveness of stroke telehealth delivery depends upon a number of factors, including trained staff, infrastructure to support CT image transfer, videoconferencing capabilities, established protocols and processes, and funding. Furthermore, effective deployment requires the coordinated training of health care staff in telestroke workflows across diverse clinical settings. TACTICS VR Stroke Telehealth was developed to address this need.

VR technology is increasingly used to support training in a range of workplace settings, including health care. VR has advantages over traditional 2D training delivery approaches in terms of presence, privacy, and immersion [53], but the relative benefit of VR regarding training outcomes depends upon the specific application as well as the delivery context. Several recent systematic reviews have collated findings on mixed reality or VR and health care staff training, with specific focuses on head-mounted devices (HMDs) [37] or augmented reality in medical education [39] and VR-based training in nursing [38]. The majority of reported studies demonstrated the effectiveness of training (or noninferiority to traditional approaches), with greater trainee enthusiasm and enjoyment when using HMD-based technology in medical education [37]; improved performance time, confidence, and satisfaction using AR for medical education [39]; and improved knowledge development using VR for nursing education [38]. However, the existing literature remains limited, with a low number of total studies (11-27 studies each across 3 systematic reviews), low total participant numbers (a total of 654-956 participants across all studies), and application to training in a limited range of medical fields (eg, 48% of HMD-based training studies in surgery and 15% in anatomy [37]). Notably, there has been limited application of VR technology for the continuing education of health care professionals, the target population in this study; for example, across all studies of HMD-based training in medical education, 60% of the study participants were medical students, and 30% were medical residents (ie, 90% combined) [37]. On the basis of the reported 956 total participants across all 27 studies, this indicates that only approximately 95 (approximately 10%) nontrainee participants were included [37]. Thus, the 177 VR sessions included in this study represent almost twice the total number of reported HMD-based VR training participants in the existing literature within a continuing medical education context. Across the 3 systematic reviews, the authors noted many limitations, including most of the studies being small-scale short-term pilot studies, often conducted in a single nonclinical setting with limited participant diversity in medical field or training stage and often poor-quality study design. Our study addressed several of these areas and provides insights into the application of VR training for the continuing education of health care professionals in a clinical context, which is a setting that is underrepresented—in terms of both population and training—in the existing literature. None of the studies identified in the systematic reviews specifically focused on the continuing education of health care professionals in stroke management. To our knowledge, the only VR applications that have been previously studied for stroke (other than our previous report of the first TACTICS VR module [24]) have targeted patient users to support poststroke rehabilitation (eg, limb motor function recovery) [35,36].

This study builds on our previous experience of developing the first TACTICS VR training module, which provided hyperacute stroke workflow training without telehealth integration [24]. The first TACTICS VR Hyperacute Stroke Management module continues to be assessed in a clustered stepped-wedge clinical trial across 3 Australian states, with data collection conducted through the end of 2022 [40]. This study differs from the previous work by upgrading content into a 6-degrees-of-freedom VR headset, developing training content specifically for stroke telehealth workflow, and assessing implementation outside of a formal structured clinical trial context. The specific elements incorporated into training include 3 telehealth consultation calls, a detailed voice-over walkthrough of CT imaging interpretation, the incorporation of an assessment support tool (termed the Acute Stroke Assessment Protocol Triage Tool), and a simulated workstation on wheels. Despite more limited infrastructure and support during implementation in this study, we noted very similar participant feedback on acceptability, feasibility, and utility, which was very encouraging. This study also builds on our previous work deploying VR training in workplaces outside of health care, which has highlighted the importance of considering broad institutional factors and developing frameworks that support training implementation, uptake, and ongoing sustainment [54-57]. Consistently across sectors (ie, in health, tertiary education [55,56], and defense [54]), these aspects must be considered and addressed at the organizational level to optimize training implementation and maximize effects.

Training programs for health care professionals in stroke management are typically delivered face-to-face by an experienced trainer or clinical expert with relevant expertise via traditional teaching modalities (eg, printed materials and slide-based presentations), informal mentor or mentee learning, and the passive distribution of guidelines and training materials. These approaches support the delivery of evidence-based clinically relevant educational content but are expensive to either deliver or scale across rural settings and are passive and limited in utility. Group education approaches contribute to relatively small improvements in professional practice (eg, <10%) [58] but are better than the passive delivery of clinical guidelines and recommendations. Limited studies have assessed simulation-based approaches to support stroke workflow training [59]. We propose that TACTICS VR training specifically and stand-alone VR-based training more broadly are a feasible and useful approach to supplement existing training modalities, particularly in rural contexts.

### Limitations

We note several limitations, which should be considered when interpreting data from this study. First, study recruitment was led by on-site staff at spoke hospitals who were requested to target health care professionals involved in stroke management; as such, it was nonrandom. This was partially mitigated by encouraging site coordinators to recruit all staff involved in stroke management. Study participants primarily included physicians and nurses in emergency or acute stroke care. We note that the demographic mix captured from TACTICS VR users in the headset was similar to that of the survey respondents in terms of department, position title, and relative stroke experience. Although this was the intended target population for this training, their feedback on the utility of VR training to support workplace training more broadly may not be representative of all health care professional groups. It may be useful to specifically target additional training populations in future studies (eg, radiographers). Second, implementation was limited to hospitals in NSW, and detailed survey collection was limited to a subset of 7 sites, with corresponding ethics approvals. This was due to the complexities in obtaining specific ethics approvals for survey distribution across multiple local health districts and hospitals. Although it would be useful to collect additional data in specific local health districts or contexts to inform the location-specific integration of training in the future, we believe that the responses collected at the 4 sites are representative of the general feedback overall because the sites included are reflective of regional Australian spoke hospitals. Third, TACTICS VR Stroke Telehealth training was deployed as part of the NSW Telestroke Service, which included additional education materials, site visits, and follow-up support, as well as staffing and quality improvement activities. VR training was intended to complement these training approaches and resources as an add-on that can be deployed on site in rural settings. However, this approach does limit the ability to directly assess and attribute the effects of TACTICS VR training on clinical behavior or patient outcomes in isolation. Metrics on clinical behavior and patient outcomes are being captured as part of the NSW Telestroke Service implementation, which will provide insights into the effects of the overall program but will not allow an assessment of the direct effect of TACTICS VR Stroke Telehealth training specifically. Fourth, given the nature of this feasibility study, no control group was included; as such, we were unable to compare the effects of TACTICS VR Stroke Telehealth training with those of alternative approaches. Finally, the survey was intentionally kept short, noting the time constraints of study participants, and limited to study-specific questions. Additional assessments, including questions relating to the implementation approach as well as the application of validated instruments, may be useful in future studies (eg, assessing presence via the Presence Questionnaire [47], usability via the System Usability Scale [46], and engagement via the User Engagement Scale [60]).

### Conclusions

User acceptance among those who used TACTICS VR Stroke Telehealth training was very high and included suggestions for future implementation and expansion. The feedback provided has directly led to the subsequent scoping and development of a dedicated stroke nursing module (TACTICS VR Stroke Nursing), and additional modules are in development to support paramedic-led stroke assessment and transport decision-making in a prehospital phase (TACTICS VR Stroke Paramedic). The platform is deemed feasible for deployment within a real-world clinical setting, supported by high ratings on perceived usability and usefulness. Further research is now required to document the effects of TACTICS VR training on clinical practice and patient outcomes as well as to assess cost-effectiveness. Further research will also be useful to optimize training uptake and identify approaches to support the integration of training into existing education pathways.

## Appendix

Multimedia Appendix 1 Supplementary tables (pretraining survey questions and responses as well as posttraining survey questions and responses).

## Abbreviations

ACI: Agency for Clinical Innovation
ATS: Centre for Advanced Training Systems
CT: computed tomography
HMD: head-mounted device
NSW: New South Wales
SME: subject matter expert
VR: virtual reality
