# Supplementary material for: TACTICS VR Stroke Telehealth Virtual Reality Training for Health Care Professionals Involved in Stroke Management at Telestroke Spoke Hospitals: Module Design and Implementation Study
Source: JMIR Serious Games. 2023 Dec 7;11:e43416. doi: 10.2196/43416 (PMC10739245; doi:10.2196/43416)
Supplement: Multimedia Appendix 1 [file games_v11i1e43416_app1.docx]

## Supplementary Materials

# TACTICS VR Stroke Telehealth virtual reality training for healthcare professionals involved in stroke management at telestroke spoke hospitals: Module design and implementation study

# Contents

**Supplementary Table 1: Pre-training survey questions and responses**

**Supplementary Table 2: Post-training survey questions and responses**

# Supplementary Tables

**Supplementary Table 1: Pre-training survey questions and responses**

| **Question** | **Response options (n)** |
| --- | --- |
| Q1: What is your location? | Telestroke hub site (5)  Rural telestroke site 1 (10)  Rural telestroke site 2 (4)  Rural telestroke site 3 (2)  Rural telestroke site 4 - 6 (0)  **Note – site names have been de-identified for public reporting due to ethics considerations* |
| Q2. What is your specialty area of work? | Emergency Care (14)  Intensive Care (1)  Radiology  Acute Stroke / Neurology (3)  Stroke Rehabilitation  Other - please specify (3):   - Stroke Dietitian (1) - Research (1) - General Acute / Medicine (1) |
| Q3. What category best describes your employment? | Nurse (10)  Doctor (10)  Nurse Practitioner  Radiographer  Other - please specify (1):   - Stroke Dietitian (1) |
| Q4: Are you currently involved in a training program (e.g. residency)? | Yes (3)  No (18) |
| Q5. Approximately how many acute stroke patients have you cared for or treated over the course of your career? (Please estimate) | Less than 10 patients (7)  11-20 patients (4)  21-30 patients (2)  31-40 patients (2)  41 patients or more (6)  Not applicable to my practice |
| Q6. Approximately how many stroke telehealth cases have you been involved in? (Please estimate) | 0 patients (8)  1-4 patients (8)  5-9 patients (0)  10-14 patients (2)  ≥15 patients (2)  Not applicable to my practice (1) |
| Q7. I am confident in my ability to effectively assess or treat acute stroke patients. | Strongly agree  Agree  Undecided  Disagree  Strongly disagree  Not applicable  (Mean = 3.52 ± 0.93, n=21; Strongly agree = 5) |
| Q8. I am confident in my ability to optimally communicate with my colleagues to enable effective treatment of acute stroke patients. | Strongly agree  Agree  Undecided  Disagree  Strongly disagree  Not applicable  (Mean = 3.71 ± 0.85, n=21; Strongly agree = 5) |
| Q9. I am confident in my knowledge around accessing telehealth for acute stroke assessment or treatment. | Strongly agree  Agree  Undecided  Disagree  Strongly disagree  Not applicable  (Mean = 3.10 ± 1.00, n=21; Strongly agree = 5) |
| Q10. I am aware of local stroke telehealth services and processes. | Strongly agree  Agree  Undecided  Disagree  Strongly disagree  Not applicable  (Mean = 3.43 ± 0.93, n=21; Strongly agree = 5) |
| Q11. I am confident in my understanding of telehealth workflow processes. | Strongly agree  Agree  Undecided  Disagree  Strongly disagree  Not applicable  (Mean = 3.19 ± 0.87, n=21; Strongly agree = 5) |
| Q12. I am likely to use telehealth services in the future | Strongly agree  Agree  Undecided  Disagree  Strongly disagree  Not applicable (1)  (Mean = 4.16 ± 0.96, n=19; Strongly agree = 5) |
| Q13. I am confident in my understanding of workflow practices to manage acute stroke patients. | Strongly agree  Agree  Undecided  Disagree  Strongly disagree  Not applicable  (Mean = 3.90 ± 0.62, n=21; Strongly agree = 5) |
| Q14. I am confident in my ability to make improvements to how acute stroke care is provided to patients presenting to this hospital. | Strongly agree  Agree  Undecided  Disagree  Strongly disagree  Not applicable  (Mean = 3.52 ± 0.87, n=21; Strongly agree = 5) |
| Q15. Have you previously used any of the TACTICS VR training modules (check all that apply)? | Yes – TACTICS VR Hyperacute Stroke Workflow (part of the TACTICS clinical trial; 3)  Yes – TACTICS VR Stroke Telehealth (current module)  No (18) |
| Q16. What is your experience with virtual reality technology? (This may include gaming and home use as well as other virtual simulation experiences – please provide as much detail as possible) | Over 100 hours of usage  50-99 hours of usage  10-49 hours of usage (1)  Less than 10 hours of usage (6)  No experience – I have never used VR or similar technology (14) |
| Q17. I believe that VR can be an effective method to teach or transfer knowledge about stroke telehealth workflow. | Strongly agree  Agree  Undecided  Disagree  Strongly disagree  Not applicable  (Mean = 3.76 ± 0.77, n=21; Strongly agree = 5) |
| Q18. I feel confident in regards to managing the technical aspect of the VR training tool. | Strongly agree  Agree  Undecided  Disagree  Strongly disagree  Not applicable  (Mean = 3.57 ± 0.75, n=21; Strongly agree = 5) |
| Q19. Are you prone to motion sickness? | Yes (2)  No (17) |
| Q19: What do you hope to learn from the TACTICS VR Telehealth Training Module? | Free-text responses:   - Test run of using VR training for stroke care coordination - Fill in gaps in telehealth workflow regarding computer & camera etc. - Unsure yet - Protocol for stroke - How to improve the delivery of care to patients that have been identified as likely had a stroke - How to assist in stroke call and learn about telehealth - How to use VR tool and any further stroke information - Ability to test my knowledge - More proficient processes in incorporating telestroke - Better able to manage stroke - Confidence in assessing acute stroke; Smooth flow of assessment processes - Acute stroke assessment and care - Improve my knowledge in assessing and managing acute stroke patients |

VR = virtual reality; Data presented as mean +/- SD.

**Supplementary Table 2: Post-training survey questions and responses**

| **Question** | **Response options (n)** |
| --- | --- |
| Q1. The VR hardware (e.g. headset and controller) were comfortable and easy to use. | Strongly agree  Agree  Undecided  Disagree  Strongly disagree  (Mean = 4.10 ± 0.91, n=20; Strongly agree = 5) |
| Q2. The TACTICS VR – Telehealth user interface was straight-forward and easy to use (e.g. menu system, buttons, etc). | Strongly agree  Agree  Undecided  Disagree  Strongly disagree  (Mean = 4.35 ± 0.67, n=20; Strongly agree = 5) |
| Q3. I enjoyed TACTICS VR - Telehealth training. | Strongly agree  Agree  Undecided  Disagree  Strongly disagree  (Mean = 4.22 ± 0.55, n=18; Strongly agree = 5) |
| Q4. TACTICS VR – Telehealth provided useful information.to be useful for trainees and staff in the management of acute stroke | Strongly agree  Agree  Undecided  Disagree  Strongly disagree  (Mean = 4.22 ± 0.43, n=18; Strongly agree = 5) |
| Q5. TACTICS VR - Telehealth provided accurate information. | Strongly agree  Agree  Undecided  Disagree  Strongly disagree  (Mean = 4.17 ± 0.71, n=18; Strongly agree = 5) |
| Q6. TACTICS VR - Telehealth was an effective tool for transferring knowledge about stroke telehealth practices. | Strongly agree  Agree  Undecided  Disagree  Strongly disagree  (Mean = 4.35 ± 0.49, n=17; Strongly agree = 5) |
| Q7. TACTICS VR – Telehealth improved my awareness of stroke telehealth approaches | Strongly agree  Agree  Undecided  Disagree  Strongly disagree  (Mean = 4.28 ± 0.57, n=18; Strongly agree = 5) |
| Q8. TACTICS VR – Telehealth improved my understanding of stroke telehealth approaches. | Strongly agree  Agree  Undecided  Disagree  Strongly disagree  (Mean = 4.17 ± 0.79, n=18; Strongly agree = 5) |
| Q9. TACTICS VR – Telehealth increased my confidence in stroke telehealth workflow. | Strongly agree  Agree  Undecided  Disagree  Strongly disagree  (Mean = 3.83 ± 0.79, n=18; Strongly agree = 5) |
| Q10. The simulation was of sufficient realism to communicate the critical aspects of workflow. | Strongly agree  Agree  Undecided  Disagree  Strongly disagree  (Mean = 3.90 ± 1.02, n=20; Strongly agree = 5) |
| Q11. The feedback provided at the end of the VR training module was constructive and useful. | Strongly agree  Agree  Undecided  Disagree  Strongly disagree  (Mean = 3.95 ± 0.60, n=20; Strongly agree = 5) |
| Q12. As a result of TACTICS VR – Telehealth training I am more likely to use stroke telehealth services in the future. | Strongly agree  Agree  Undecided  Disagree  Strongly disagree  (Mean = 4.00 ± 0.75, n=19; Strongly agree = 5) |
| Q13. Did you feel motion sick or nauseous while participating using TACTICS VR - Telehealth? | Yes – I could not compete the training due to motion sickness  Yes – But I was able to complete training (3)  No (17) |
| Q14: What elements of the TACTICS VR – Telehealth training module were most beneficial? | Free-text responses:   - Visual environment & scans; feedback on selections made; step by step process - Explanation of Telestroke workflow & input - Easy to follow - Instant feedback – awesome - Clear concise info - Easy to answer questions – good format - Realistic, easy to use, understandable - Running through sequence of processes - Overview of flow process - Realism - Better understanding of imaging and rationale - Explanations provided at each decision point - Being guided through the process - Candidate to decide management plan; when to call telestroke team - Being able to appreciate patient flow; moving from ED to imaging etc - Feedback / interaction |
| Q15: What elements of the TACTICS VR –Telehealth training module could be improved? | Free-text responses:   - Perhaps the selections could be more clear regarding only selecting the first step in parallel ie. checklist that you tick - Need pause button - I was a bit unsure what to do going through motions or checking every box - Role-oriented – not nurse-specific seems more medical officer - Some assumed knowledge of abbreviations specific to stroke - Set up same as computer video learning but in 3D – not making the most of VR. Could get patient / staff to talk to you and allow me to make more decisions rather than click boxes - Mock scenario at beginning or instructions on how to navigate through scenarios - Didn’t understand scoring system at end - Quicker, quite slow - Ease of setup - ED processes are incorrect – cannot gain personal details over the bat phone with ambulance, and patient cannot stay on ambulance stretcher due to 30-minute KPI – Patient always offloaded straight to resus bay - Would be useful to practice performing NIHSS in a more detailed manner - Nil - Looking for people to run the process; I could not see the radiographer next to me - delay management - Further info regarding interpreting scans; Include information re: contraindications to tPA / ECR - Lighter headset; content is very good |
| Q16: Should additional modules for TACTICS VR stroke training be developed?  If yes, what modules would be most useful? | Yes (13)  No (5)  Free-text responses:   - Allied health team involvement - or physio, speech, dietitian to demonstrate whole of team care - A separate module for JMOs in ASAP assessment to decrease inter-operator variability - Trauma survey, cardiac thrombolysis - Time critical information - Procedural skills training - Critically ill patient; NSTEMI / STEMI, GI bleeding - Imaging interpretation; How to access telestroke services in rural settings |
| Q17: Did you experience any issues that prevented you from completing TACTICS VR – Telehealth training?  If yes, please describe: | Yes (0)  No (19)  Free-text responses:   - *No issues reported* |
| Q18. I am confident in my ability to optimally communicate with my colleagues to enable effective treatment of acute stroke patients. | Strongly agree  Agree  Undecided  Disagree  Strongly disagree  Not applicable to my practice  (Mean = 4.22 ± 0.55, n=18; Strongly agree = 5) |
| Q19. I am confident in my knowledge around accessing telehealth for acute stroke assessment or treatment. | Strongly agree  Agree  Undecided  Disagree  Strongly disagree  Not applicable to my practice  (Mean = 4.11 ± 0.58, n=18; Strongly agree = 5) |
| Q20. I am aware of local stroke telehealth services and processes. | Strongly agree  Agree  Undecided  Disagree  Strongly disagree  Not applicable to my practice  (Mean = 4.11 ± 0.58, n=18; Strongly agree = 5) |
| Q21. I am confident in my understanding of telehealth workflow processes. | Strongly agree  Agree  Undecided  Disagree  Strongly disagree  Not applicable to my practice  (Mean = 4.22 ± 0.55, n=18; Strongly agree = 5) |
| Q22. I am confident in my understanding of workflow practices for acute stroke assessment and treatment. | Strongly agree  Agree  Undecided  Disagree  Strongly disagree  Not applicable to my practice  (Mean = 4.28 ± 0.46, n=18; Strongly agree = 5) |
| Q23. I am confident in my ability to make improvements to how acute stroke care is provided to patients presenting to this hospital. | Strongly agree  Agree  Undecided  Disagree  Strongly disagree  Not applicable to my practice  (Mean = 4.00 ± 0.59, n=18; Strongly agree = 5) |

VR = virtual reality; Data presented as mean +/- SD.
